# Editorial: Women in translational pharmacology: 2021

**DOI:** 10.3389/fphar.2023.1162722

**Published:** 2023-02-20

**Authors:** Valentina Vengeliene, Lu Qin

**Affiliations:** ^1^ Department of Neurobiology and Biophysics, Institute of Biosciences, Life Sciences Center, Vilnius University, Vilnius, Lithuania; ^2^ Heart and Vascular Institute, The Pennsylvania State University College of Medicine, Hershey, PA, United States

**Keywords:** translational pharmacology, female researchers, global health, female health, gender equality

## Introduction

Women in Translational Pharmacology:2021 is part of a series of article Research Topic hosted throughout Frontiers in Pharmacology in response to the long-standing biases and gender stereotypes discouraging girls and women from science-related fields. At present, according to **UNESCO**, less than 30% of researchers worldwide are women. The field of translational pharmacology is also impacted in several other ways, including the underrepresentation of female experimental animals in preclinical research (Lee, 2018), and the likelihood of inclusion of gender and sex analysis in the research. This issue has been recognized by the NIH, which since 2016 requires sex to be considered a biological variable (Arnegard et al., 2020) and to provide a strong rationale for any single-sex studies. Similarly, since 2020 European Commission requires grant recipients to incorporate sex and gender analyses into the design of research studies (Nature, 2020). In 2017, Nielsen et al. (2017) showed that simply promoting the scientific advancement of women positively influenced the likelihood of inclusion of gender and sex analysis in the research.

This Research Topic received submissions showing both the work of female researchers and work focusing on female-dominant diseases in a Research Topic of five original and review articles.

## Summary of the articles in this Research Topic

The contribution from Fardoun et al. introduced us to an innovative concept of using G-coupled protein estrogen receptor (GPER) as the therapeutic target for Raynaud’s phenomenon (RP), a pathological condition caused by the hyperactive cold-induced vasoconstriction that is more prevalent in females than males. The authors proposed a pathway in how activation of GPER upregulates the expression of vascular alpha 2C-adrenoceptors (α_2C_-AR).

The literature review by Yang et al. discussed the role of melatonin in the development and the treatment of postmenopausal osteoporosis. The authors provided an overview of research findings regarding the correlation between the level of serum melatonin and bone mass, and the effect of melatonin on bone remodeling for promoting osteogenesis and suppressing osteoclastogenesis. As bone mass loss is one of the chief health concerns for the menopause population, this review brought an insightful perspective on utilizing melatonin as an alternative treatment strategy for postmenopausal osteoporosis.

In the original article from van de Vyver et al., the researchers established an experimental protocol to investigate pharmacokinetic processes of brain uptake of a 195 kDa monoclonal antibody EGFRvIII-TCB in healthy rats after intravenous (IV) or intracerebroventricular (ICV) administration. The findings of this study may facilitate cross compound comparison, which the authors demonstrated by comparing EGFRvIII-TCB with two other tool compounds.

The literature review by Arip et al. provided an oversight of the known resistances in bacteria, viruses, fungi and parasites and the remaining traditional treatment options including a discussion of the limitation of the current therapeutic approaches. The authors also presented an overview of several plant-based compounds, their mechanism of action and the potential in tackling antimicrobial resistance (AMR). We hope that readers of this article can benefit from the authors’ knowledge in understanding the feasibility of utilizing plant-based metabolites and generate new ideas for research to tackle this global concern.

The study by Huang et al. investigated the effect of two Chinese medicines, chitosan and danshen, on obstructed fallopian tubes, which is one of the main causes for reduced fertility among women. Chitosan is a non-toxic extract from the shells of marine creatures and danshen comes from the dried roots and rhizomes of Salvia miltiorrhiza Bge. This study showed that the effective constituent of chitosan and danshen injection was stable, effective at preventing tubal re-obstruction and increased pregnancy rates.

We, as an editorial team, sincerely appreciate the authors’ willingness and enthusiasm to contribute their research stories to this topic. It is our hope that the number of female researchers will continue to grow in the future and that the research community will invest more effort in conditions impacting women.
